# Radiographic Evaluation of Impacted Third Mandibular Molar According to the Classification of Winter, Pell and Gregory in a Sample of Cameroonian Population

**DOI:** 10.4314/ejhs.v33i5.15

**Published:** 2023-09

**Authors:** Edouma Jacques, Messina Ebogo, Yann-Chris Eng, Ntenkeu Donald, Zeh Odile

**Affiliations:** 1 Department of parodontology, oral and maxillofacial surgery, Faculty of medicine and biomedical Sciences, University of Yaounde I, Yaounde-Cameroon; 2 Department of oral and maxillofacial surgery, Cheikh Anta Diop University of Dakar, Dakar-Senegal; 3 Department of radiotherapy, radiology and medical imaging, Faculty of medicine and biomedical Sciences, University of Yaounde I, Yaounde-Cameroon

**Keywords:** mandibular 3^rd^ molar, panoramic radiography, Winter classification, clinical landmarks

## Abstract

**Background:**

The extraction of impacted third molars (M3) is a common surgical procedure in dentistry and oral surgery. Various complications, including inferior alveolar nerve (IAN) damage, may occur during and after extraction of this tooth. Radiographic examination should provide information about the M3 itself, but also about the surrounding bony structure and the relationship of the roots to the IAN and the adjacent second molar, which is often traumatized during this extraction. The aim of our study was to evaluate the depth and angulation of impacted mandibular third molars (M3) from panoramic radiographs, according to the classifications proposed by Winter and Pell & Gregory.

**Methods:**

Radiographic signs present on the orthopantomogram showing M3 depth, and retromandibular available space according to the Pell & Gregory classification were evaluated. Evaluation of the M3 angulation relative to the M2 according to Winter's classification was also done. Student's t test was used to determine the association between side or sex and different variables.

**Results:**

The depth of impaction of the M3 crown was level A accounting for 54.4% (n=260) of the PR while level B constituted 35.7% (n=171) of the images. Regarding the availability of retromandibular space, Class I constituted 36.8% (n=176). The Class II accounted for 55.9% (n=267) of PR.

**Conclusion:**

Our study showed that 54.4% of M3 were located at the same level as the occlusal plane of the second molar, while in 56% of PR the space between the second molar and the ramus of the mandible is less than the mesiodistal diameter of the third molar. This research showed that 23.1% of M3 had a level of vertical angulation, a level that allows for less painful luxation of the impacted molars. These results seem to show a relatively high level of difficulty in mobilizing and extracting M3 from Cameroonian patients.

## Introduction

The lack of knowledge of the thickness of the alveolar bone in different areas of the tooth can lead to an inappropriate extraction protocol and, consequently, often long-term intraoperative and postoperative complications. The incidence of inferior alveolar nerve (IAN) lesions after impacted mandibular third molar (M3) extraction has ranged from 0.4% to 13.4% (1). However, in most cases, the sensory disturbances are reversible within a few weeks to months. Less than 1% of patients will have permanent damage to the IAN (2). Although several factors may influence the occurrence of IAN lesions, a major risk factor is thought to be the proximity of the M3 to the IAN (3). The overall risk of lingual nerve injury is between 0.5 and 2.6%. The risk of lingual nerve injury is estimated by some authors to be as low as 0.5-2.6% and as high as 6.6% in some individuals (4). However, the results of most studies have suggested that the loss of sensation will be temporary and will resolve within a few months after extraction (5). Renton and McGurk (4) reported that the position of the M3 and the depth of this tooth can be evaluated radiologically to assess the level of difficulty during extraction.

Assessing the angulation of the M3 is an essential t ool for planning the extraction of the M3, as it enables us to predict the degree of operative difficulty, and to plan the surgical equipment required, as well as the operating time (5). The difficulty of M3 surgery can be determined from radiographic, anatomic, demographic, and operative parameters (6). Radiographic criteria remain the indicators of choice for assessing the difficulty of M3 surgery. Typically, conventional 2-dimensional (2D) images, such as panoramic radiographs, will be used as the standard means of preoperative exploration of the third molar extraction and its relationship with the surrounding structures (7).

The aim of this study was to evaluate, using panoramic radiographs, the depth of inclusion of M3. We also determined the angulation of the impacted mandibular third molars. Angulation and depth of inclusion were determined according to the classifications proposed by Winter and Pell & Gregory.

## Material and Methods

**Study design**: This was a descriptive cross-sectional study on panoramic radiographs. The research took place over a 20-month period, from March 2020 to November 2021.

**Setting**: This study was carried out at the oral implantology Department of Yaounde's Faculty of medicine and biomedical Sciences in Cameroon.

**Impacted third mandibular molar (M3) definition**: For a M3 to be considered impacted, it must meet two conditions: the roots of the third molar are fully formed, except for horizontally or transversely impacted molars; and there is no functional occlusion on the occlusal surface of the third molar.

**Participants**: For this research, panoramic radiographs of patients with mandibular molars in the mouth and clearly showing M3 and its relationship with surrounding structures were included in this work. Panoramic radiographs from patients at least 18 years of age with adequate diagnostic image quality showing complete apexification of M3 and intact bone in the posterior mandibular area were selected for our study. We selected 304 panoramic radiographs from individuals meeting our inclusion criteria.

**Data source/measurement**: The analysis of the radiographic images was carried out using pantographic radiographs taken digitally with a camera Cranex3 Dx with a magnification of 1:1.19. Analysis of the digital images provided by the pantograph was done using the RayScan program 5.2.6

**Bias**: Before analyzing our results, we checked the reproducibility of the measurements and the inter- and intra-observer measurements. For this purpose, 20 panoramic radiographies (PR) were selected, taking into account gender and side, by a second operator who had performed a similar Cone beam CT study and had several years of experience in craniometric studies. Cohen's kappa calculation was used to measure the agreement between our results and those obtained by an independent radiologist (8).

**Study size**: A non-probability, non-randomized study was conducted. Panoramic radiographies were included in the study according to the inclusion criteria they met.

**Study variables**: The variables studied were the location, depth and angulation of the mandibular third molars, according to the classifications proposed by Winter and Pell & Gregory (9). The level of impaction was determined using Pell and Gregory classification. Pell and Gregory classification is another system that is widely used to access the degree of difficulty of extracting M3.

**The Pell and Gregory classification (9)**: The depth of impaction of the crown of the M3 was considered in relation to occlusal plane of the adjacent second molar:

**Level A**: The occlusal plane of the impacted tooth is at the same level as the occlusal plane of the second molar;

**Level B**: The occlusal plane of the impacted tooth is between the occlusal plane and the cervical line of the second molar;

**Level C**: The impacted tooth is below the cervical line of the second molar.

**Figure 1:**
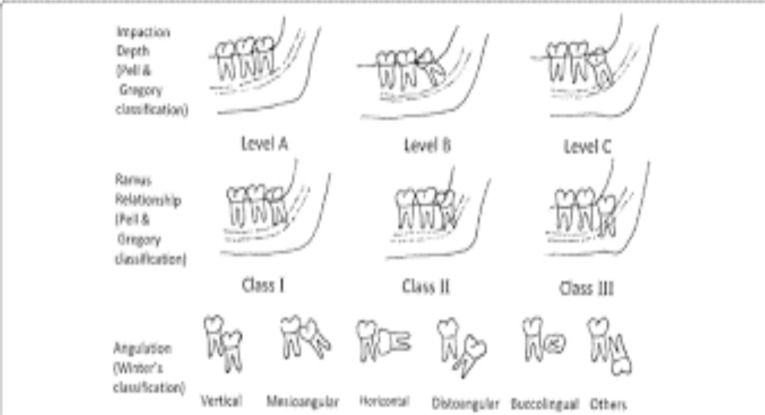
Pell & Gregory classification (9)

The relation of the tooth to the anterior border of the ramus of mandibular second molar is classified as below (9) :

**Class I**: There is sufficient space between the ramus and the distal part of the second molar for the accommodation of mesiodistal diameter of the third molar;

**Class II**: The space between the second molar and the ramus of the mandible is less than the mesiodistal diameter of the third molar;

**Class III** : All or most of the third molar is in the ramus of the mandible (Figure 1).

The mandibular third molar was also studied according to Winter's classification with reference to the angle formed between the lines intersecting the long axis of the second and third molars (10).

**Classification according to angulation (the Winter Classification) (11)**:

- Vertical impaction: 10° to -10°
- Mesioangular impaction: 11° to 79°
- Horizontal impaction: 80° to 100°
- Distoangular impaction: -11° to -79°

**Figure 2:**
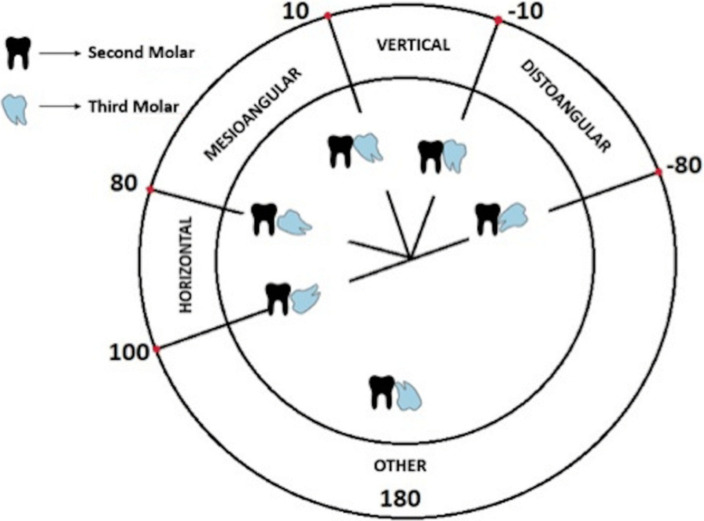
Angulation of M3 according to Winter classification (11)

Less frequent angulations such as buccolingual, mesio-inverted, disto-inverted, and disto-horizontal angulations were classified as “other” (Figure 2) (11).

**Statistical analysis**: The data from our sample were recorded in a data entry mask at the same time as the collection process. Processing of the collected data was done using SPSS version 20.1 software according to our variables. Mean and median were calculated as indicators of central tendency, and standard deviation and interquartile range were calculated as indicators of variability, along with their confidence intervals. The p<0.05 value was considered statistically significant.

Student's t-test was used in our study for the analysis of inter- and intra-observer reproducibility at the 5% threshold. The p-values found were between 0.21 and 0.54. These values are above the significance level, implying that our study is 95% reproducible. Descriptive analysis of the results was carried out, and a comparison of the variables studied according to gender and side was made using Student's t-test. The results were then compared with data reported in the literature, using comparisons of means.

**Ethical considerations**: The research protocol was submitted to the Institutional Ethics and Research Committee of the Faculty of Medicine and Biomedical Sciences of Yaoundé for approval. The anonymity of the data was respected through out our research. The authorization of the head of the Dental Radiology and Implantology Laboratory was obtained prior to the collection of data for our survey.

## Results

**Participants**: In this research, 478 panoramic radiographs that met our inclusion criteria and thus represent 956 mandibular third molars studied. No statistically significant difference between the variables studied and either gender or side was found. Female patients represented 52.9% (n=253) of our participants.

**Main results**: The depth of impaction of the M3 crown was level A accounting for 54.4% (n=260) of the panoramic radiographs while level B constituted 35.7% (n=171) of the images. Regarding the availability of retro-mandibular space, Class I constituted 36.8% (n=176). The Class II accounted for 55.9% (n=267) of PR (Table 1). In our cohort, 15.2% (n=73) of PR had M3 in horizontal position while 23.1% (n=110) of M3 were mesioangular, and 5.9% (n=28) were distoangular (Table 2)

**Table 1:** the depht and position of M3 according to Pell & Gregory classification

Depth of impaction of the M3 crown	Right side	Left side
Level A	260 (54.4%)	250 (52.4%)
Level B	171 (35.7%)	152 (31.8%)
Level C	47 (9.9%)	76 (15.8%)
**Retro mandibular available space**	**Right side**	**Left side**
Class I	176 (36.8%)	202 (42.2%)
Class II	267 (55.9%)	244 (51%)
Class III	35 (7.3%)	32 (6.8%)

**Table 2:** Angulation of the M3 according to Winter classification

Impaction Class	Right side	Left side
Horizontal	93 (19.6%)	73 (15.2%)
Vertical	250 (52.2%)	256 (53.5%)
Mesioangular	96 (20.1%)	110 (23.1%)
Distoangular	30 (6.3%)	28 (5.9%)
Others	9 (1.8%)	11 (2.3%)

## Discussion

The extraction of M3 is a fairly frequent act in consultation of oral surgery and dentistry. It requires a good diagnosis and a well-conducted radiographic exploration for optimal management of this extraction. Some tools like classifications of the spatial location of the impacted lower third molars allow us to determine the depth and angulation of impaction, which allows to determine preoperatively the degree of difficulty of the procedure and to predict possible complications of the act (12). In the literature, authors most often use the classifications of Winter, Tetsch and Wagner, Pell and Gregory, and Asanami and Kasazaki (13).

Winter's classification is the most commonly chosen method for the spatial evaluation of teeth included in the literature due to its simplicity of use. It does not require the use of additional measuring instruments, which influences its widespread use in clinical practice. In our study, the most common degree of depth of M3 impaction was level A (54.40%). Regarding the anterior edge of the mandibular ramus, the impacted molar was most often in position 2 (55.9%). Some Iranian researchers have classified the degrees of impaction according to Pell and Gregory (14). In their study, they evaluated 1,165 impacted third molars, of which 64.4% were at level A depth. In their samples, class 2 was also the most found. The most common impaction types in the study were 2A (38.93%) and 2B (17.67%), and the least common was 3A (1.87%). However, our results remain partially different from those of Eshghpour et al (15) who reported a level B (63.85%) and class 2 in (42.46%) of the individuals included in his sample. In a study conducted on Chinese populations in Singapore, Quek et al (16) had also found a predominance of level B (85%) of presentations. El Khateeb et al (17) found an equal distribution of class B and A (44.8%) over the 458 PR studied in a sample of Saudi population. According to the Demirel et al (18) in a Turkish population, the Level C was the most represented and class 2 the most found. Of 230 PR included in the study of Hatem et al (19), 66.7% presented a M3 in level C and class III. This finding is different from our research.

The most frequent type of impaction was vertical impaction (52.2%) while mesioangulation accounted for 23.1% of PR according to Winter classification. Padhye et al (20) presented an analysis of 1200 orthopantomographs, in which 33.33% of the subjects had a mesial angle alignment according to Winter. Kumar et al (21) observed the prevalence of mesial angle alignment in 52.89% of cases in Eritrean residents. In the studies of Al-Dajani et al (22) and Yilmaz et al (23), vertical impaction was found to be the most frequent position. The first team showed the presence of this impaction in 40.7% of cases and mesioangular impaction in only 7.1% of patients; the second team showed vertical impaction in 53% of cases and mesioangular impaction in 29% of cases. The differences in results may be due to the adoption of an incorrect modification of Winter's index in the studies by Al-Dajani et al (21) and Yilmaz et al (23).

The researchers determined the long axes of the second and third molars to determine the angulation of the impacted molar. The reference point for the measurements was the axis of the second molar. The angle of deviation of the wisdom tooth axis from the second molar axis was measured. If the deviation from the reference line was 10° on either side, the tooth impaction was defined as vertical. It may appear that this modification is identical to the angles adopted in the classification of Tetsch and Wagner. However, Al-Dajani (21) and Yilmaz (23) measured the magnitude relative to the second tooth axis, in contrast to the classification of Tetsch and Wagner, which measures the angle between the occlusal plane and the axis of the included tooth (16). Researchers from Hong Kong (24) presented horizontal impaction as the most common type. In their study of 7486 patients, 42.45% of whom had impacted lower wisdom teeth, they showed the presence of horizontal impaction in 47.45% of the patients analyzed. Horizontal impaction of the left maxillary third molar, vertical impaction of the right maxillary third molar and the left mandibular third molar are rare, but were found in a 30-year-old patient (25). In our study, the second most common impaction (39.04%) was distal-angular alignment. Carter (6) and Goyal et al (26) found that mesioangular impaction was significantly more prevalent than other forms of impaction followed by vertical impaction.

Panoramic radiography is an essential tool for programming and planning dental extraction. It allows an overview of the wisdom tooth, its depth and its angulation in order to assess the various difficulties related to the extraction of the M3. This study showed that 54.4% of M3 were located at the same level as the occlusal plane of the second molar, while in 56% of PR the space between the second molar and the ramus of the mandible is less than the mesiodistal diameter of the third molar. This research showed that 23.1% of M3 was vertically angulated; a level that allows less painful luxation of the impacted molars. These results seem to show a relatively high level of difficulty in mobilizing and extracting M3 from Cameroonian patients. However, buccolingual position and dimension cannot be assessed from a 2D radiograph such as the panoramic radiography used in the present study.
